# Impaired antibody response to COVID‐19 vaccination in patients with chronic myeloid neoplasms

**DOI:** 10.1111/bjh.17644

**Published:** 2021-06-24

**Authors:** Onima Chowdhury, Hannah Bruguier, Garry Mallett, Nikolaos Sousos, Kirsty Crozier, Caroline Allman, David Eyre, Sheila Lumley, Marie Strickland, Christina S. Karali, Lauren Murphy, Alex Sternberg, Katie Jeffery, Adam J. Mead, Andy Peniket, Bethan Psaila

**Affiliations:** ^1^ MRC Molecular Haematology Unit MRC Weatherall Institute of Molecular Medicine University of Oxford Oxford UK; ^2^ National Institute of Health Research Oxford Biomedical Research Centre Oxford UK; ^3^ Department of Haematology Churchill Hospital Oxford University Hospitals’ NHS Foundation Trust Oxford UK; ^4^ Nuffield Department of Population Health Big Data Institute University of Oxford Oxford UK; ^5^ Department of Microbiology Oxford University Hospitals’ NHS Foundation Trust Oxford UK; ^6^ Nuffield Department of Medicine University of Oxford Oxford UK

The COVID‐19 pandemic has had a severe impact on people with blood cancers. Patients with blood cancer are at an increased risk of poor outcomes after COVID‐19 infection, with more severe infections and a higher case fatality rate,^1^, ^2^, ^3^ which may reflect immunocompromise due to their underlying disease and/or immunosuppressive treatments. As a result, regulatory approval and the subsequent rapid roll out of COVID‐19 vaccines has been extremely well received by the blood cancer community. However, the registry trials of the currently approved vaccines largely excluded patients with blood cancers. Immune responses in this diverse group therefore require urgent study to ensure that these vulnerable patients are adequately protected from severe COVID‐19 infection.

A recent study demonstrated substantially reduced seroconversion rates post‐COVID‐19 vaccination in oncology patients, when compared to healthcare worker (HCW) controls, following a single dose of the Pfizer‐BioNTech BNT162b2 vaccine. Response rates were >90% in HCWs but <40% in patients with solid cancers, and strikingly low (<15%) in the 56 patients studied with haematological malignancy.^4^ Impaired immunity and reduced seroconversion are unsurprising in a heavily treated patient group, those with aggressive disease, marked cytopenias, and those with B‐cell neoplasms. This study only included three patients with myelodysplastic syndrome (MDS)/myeloproliferative neoplasms (MPNs) and myelofibrosis (MF) and none with chronic myeloid leukaemia (CML). Responses in patients with chronic myeloid neoplasms, many of whom are minimally treated and have normal or only mildly deranged blood counts, might be expected to be less severely impaired, and therefore warrant specific study.

We therefore sought to gather real‐world data by measuring anti‐SARS‐CoV‐2 IgG spike (S) antibody levels, more than two weeks following a single dose of either BNT162b2 or the AstraZeneca‐Oxford ChAdOx1 nCoV‐19 (AZD1222) vaccines in patients with CML, essential thrombocythemia (ET), polycythaemia vera (PV), MF and MDS attending the Churchill Hospital Myeloid Clinic, Oxford University Hospital, UK. Patients with clinical or laboratory evidence of prior COVID‐19 infection were excluded from the study. As part of routine clinical blood sampling, quantitative anti‐S antibody titres were measured using the EU compliant (CE‐marked) Abbott SARS‐CoV‐2 IgG II Quant Assay (Maidenhead, UK) (positive threshold: ≥ 50 AU/ml) and anti‐nucleocapsid (N) antibodies (indicative of past infection) were measured using the Abbott SARS‐CoV‐2 IgG assay. Leucocyte immunophenotyping (T/B/NK cell subsets) and serology to other previous immunizations (tetanus and pneumococcus) were also measured. Deidentified data on HCWs was used as a comparative cohort, obtained from the Infections in Oxfordshire Research Database (Research Ethics Committee, Health Research Authority and Confidentiality Advisory Group approvals: 19/SC/0403, 19/CAG/0144).

Between 1st January and 30 April 2021, samples were collected on 60 myeloid cancer patients with no prior clinical or laboratory evidence of prior COVID‐19 infection (CML, *n* = 12; ET, *n* = 17; MF/pre‐fibrotic MF, *n* = 7; PV, *n* = 11 and MDS, *n* = 13; Table  I). One patient with ET (ID 19) was subsequently found to have anti‐N antibodies indicative of past COVID‐19 infection, and was therefore excluded from further analysis. The median age of the cohort was 62 [interquartile range (IQR) = 52–73], with a similar number being male (*n* = 27) and female (*n* = 32). The majority were of white ethnicity, reflecting the local patient population. Seventy‐one percent (42/59 patients) were on active therapies, including hydroxycarbamide (*n* = 11), pegylated interferon (*n* = 8), ruxolitinib (*n* = 4), hydroxycarbamide and ruxolitinib (*n* = 1), BET inhibitor (*n* = 1) and tyrosine kinase inhibitors (TKI; imatinib *n* = 6; dasatinib *n* = 2; nilotinib *n* = 2; bosutinib *n* = 2). MDS patients were receiving azacitidine (*n* = 3), erythropoietin (*n* = 3) or an IDH1 inhibitor (*n* = 1). Twenty‐nine percent of patients (17/59) were not receiving any cytoreductive treatments or TKI. The median interval between vaccination and anti‐S antibody measurement was 34 days (IQR 28–56).

**Table I:** Demographics and clinical characteristics of patient cohort with serological response.

ID	Age	Sex	AZ or Pfizer	No. days between vaccine and anti‐S testing	Anti‐S titre (AU/ml)	Anti‐S positive or negative	Diagnosis	Current cytoreductive treatment/TKI
1	72	F	AZ	28	16·8	Neg	CML	Imatinib
2	62	F	AZ	28	54·2	Pos	CML	Imatinib
3	40	F	AZ	46	111·1	Pos	CML	Imatinib
4	53	F	Pfizer	28	257·8	Pos	CML	Imatinib
5	52	M	AZ	28	90·2	Pos	CML	Imatinib
6	47	M	AZ	56	559·5	Pos	CML	Imatinib
7	60	F	AZ	31	104·9	Pos	CML	Nilotinib
8	31	M	AZ	67	375·2	Pos	CML	Nilotinib
9	67	M	AZ	28	31·1	Neg	CML	Bosutinib
10	35	F	AZ	34	354·6	Pos	CML	Bosutinib
11	47	M	AZ	31	49·3	Neg	CML	Dasatinib
12	52	F	AZ	31	3428	Pos	CML	Dasatinib
13	77	F	AZ	26	195·1	Pos	ET	Anagrelide
14	79	F	Pfizer	68	19·9	Neg	ET	Hydroxycarbamide
15	76	F	Pfizer	47	41·8	Neg	ET	Hydroxycarbamide
16	54	M	AZ	38	2·4	Neg	ET	Hydroxycarbamide
17	69	M	AZ	55	14·10	Neg	ET	Hydroxycarbamide
18	72	M	Pfizer	28	961·2	Pos	ET	Hydroxycarbamide
*19*	*49*	*F*	*Pfizer*	*8*	*19 538·2*	*Pos*	*ET*	*Hydroxycarbamide*
20	57	M	Pfizer	68	393·7	Pos	ET	Hydroxycarbamide
21	67	M	AZ	24	4·8	Neg	ET	Interferon
22	52	M	Pfizer	35	828·7	Pos	ET	Interferon
23	48	F	Pfizer	55	328·1	Pos	ET	Interferon
24	47	F	AZ	34	134·3	Pos	ET	Interferon
25	48	F	AZ	83	68·2	Pos	ET	Interferon
26	41	M	AZ	7	1944·7	Pos	ET	None
27	53	F	AZ	41	111·60	Pos	ET	None
28	49	F	AZ	82	110·8	Pos	ET	None
29	54	F	AZ	21	12·7	Neg	ET	Ruxolitinib
30	55	F	AZ	32	5·9	Neg	PV	Hydroxycarbamide
31	71	F	AZ	60	41·7	Neg	PV	Hydroxycarbamide
32	58	F	AZ	65	37·5	Neg	PV	Hydroxycarbamide
33	56	M	Pfizer	41	242·8	Pos	PV	Hydroxycarbamide
34	70	M	AZ	27	1758·9	Pos	PV	Hydroxycarbamide
35	51	M	Pfizer	30	793·6	Pos	PV	Interferon
36	54	M	AZ	31	209·5	Pos	PV	Interferon
37	71	F	Pfizer	28	6·7	Neg	PV	Ruxolitinib
38	65	M	AZ	47	4·9	Neg	PV	Ruxolitinib
39	85	F	Pfizer	76	27·7	Neg	PV	Venesection
40	81	M	Pfizer	73	2374·6	Pos	PV	Venesection
41	62	M	Pfizer	50	152·5	Pos	MF	BET inhibitor
42	79	F	AZ	48	308·9	Pos	MF	Darbopoietin
43	51	F	AZ	47	159·2	Pos	MF	None
44	69	F	AZ	31	22·9	Neg	MF	None
45	70	M	AZ	65	3·1	Neg	MF	Ruxolitinib + hydroxycarbamide
46	74	M	AZ	23	46·8	Neg	MF	Ruxolitinib
47	48	F	AZ	32	1406	Pos	Pre‐fibrotic MF	Interferon
48	74	M	AZ	74	8·1	Neg	MDS	Azacitidine
49	69	F	Pfizer	69	5·7	Neg	MDS	Azacitidine
50	74	M	AZ	28	51·6	Pos	MDS	Azacitidine
51	57	M	AZ	14	75·3	Pos	MDS	Darbopoietin
52	86	M	Pfizer	60	55·3	Pos	MDS	Darbopoietin
53	76	M	Pfizer	21	1·7	Neg	MDS	None
54	58	F	AZ	66	4255	Pos	MDS	None
55	88	M	Pfizer	20	47·7	Neg	MDS	None
56	76	F	Pfizer	45	4·8	Neg	MDS	Transfusions
57	83	F	Pfizer	15	7·6	Neg	MDS	Transfusions
58	82	F	Pfizer	56	1345·3	Pos	MDS	Transfusions
59	72	F	Pfizer	29	159·2	Pos	MDS	Transfusions
60	73	M	Pfizer	17	19·1	Neg	MDS	IDH1 inhibitor

Patient 19 (italic font) was found to have positive anti‐N antibodies indicative of past COVID‐19 infection, and was excluded from subsequent analysis. CML, chronic myeloid laukaemia; ET, essential thrombocythemia; PV, polycythaemia vera; MF, myelofibrosis; MDS, myelodysplastic syndrome; TKI, tyrosine kinase inhibitors.

Serological responses >14 days after a single dose of BNT162b2 or AZD1222 vaccine in HCWs (i.e. predominately healthy adults) with no clinical or laboratory evidence of past COVID‐19 exposure have been reported at almost 100% (2706/2720 [99·5%] after BNT162b2 and 864/890 [97·1%] following AZD1222).^5^ As a comparative control group for our patient cohort, we collated the serological responses from a large cohort of 232 HCWs aged >60 years [median age 62 (range 60–76)]. In HCWs of a comparable age to our patient cohort, seroconversion rates were also extremely high [166/169 (98%) and 58/63 (92%) for BNT162b2 and AZD1222 respectively], >14 days following the first vaccine dose (Fig 1A,B).

**Fig 1:**
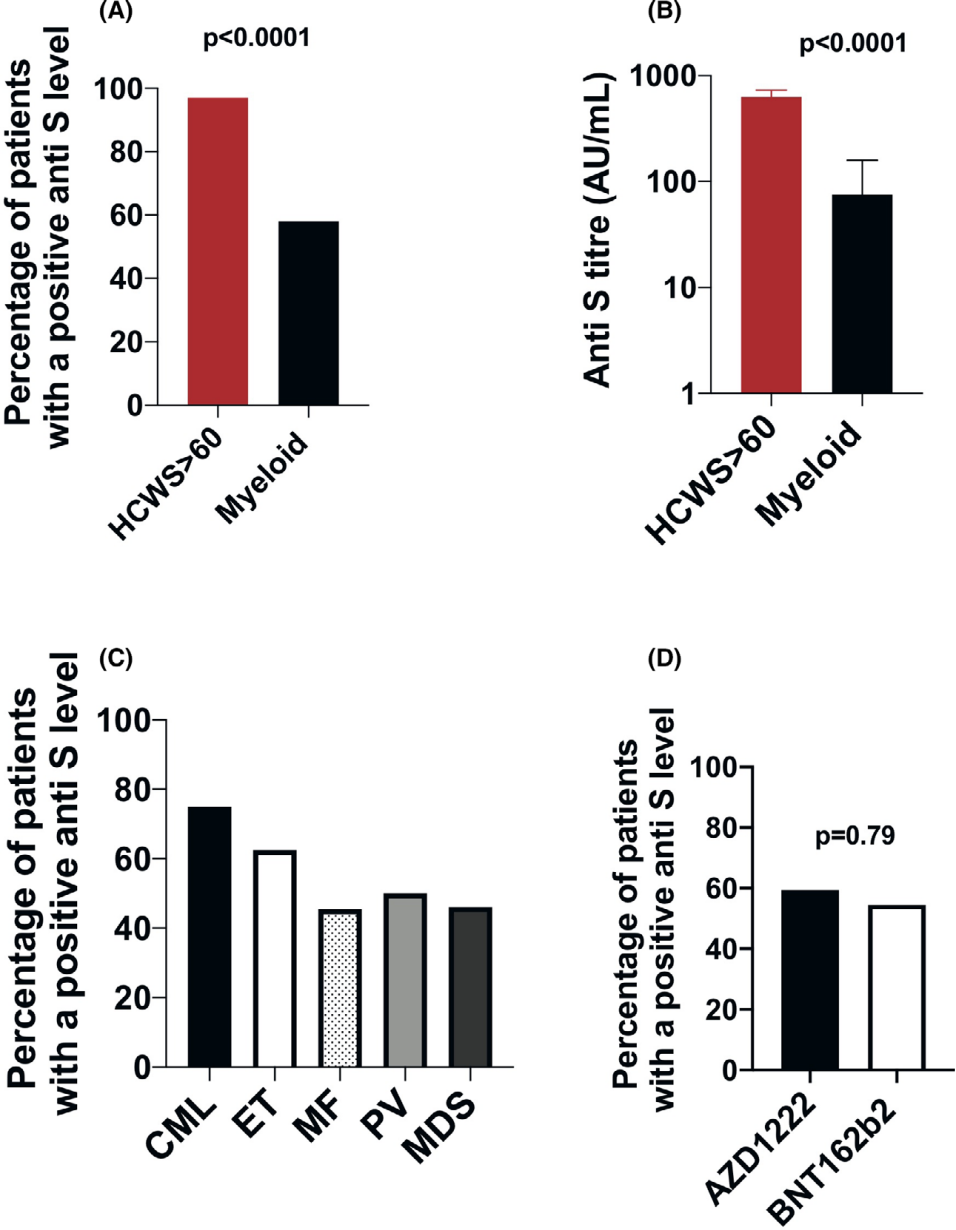
Seroconversion of patients with chronic myeloid neoplasms compared to controls post a single COVID‐19 vaccination. (A) Percentage of patients with a chronic myeloid malignancy (*n* = 59) *versus* healthcare worker (HCW) controls (*n* = 232) of a similar age (>60 years), with positive anti‐spike (anti‐S) serology and no evidence of past COVID‐19 infection [224/232 (97%) HCW vs 34/59 (58%) patients; *P* < 0·0001]. (B) Median anti‐S titre (AU/ml) after a single dose of vaccine in HCWs aged >60 years and in patients with a chronic myeloid malignancy {HCWs 630 [interquartile range (IQR) 284–1 328] vs. myeloid 75 (IQR 19–328); *P* < 0·0001}. (C) Percentage patients with positive anti‐S serology according to disease subtype: chronic myeloid leukaemia (9/12 = 75%), essential thrombocythemia (ET 10/16 = 63%), polycythaemia vera (PV 4/11 = 46%), myelofibrosis (MF 3/6 = 50%) and myelodysplastic syndrome (MDS 6/13 = 46%). (D) Percentage patients with positive anti‐S serology according to vaccine received (AZD1222 19/31 [59%] vs. BNT162b 212/21 [55%]; *P* = 0·79). Mann–Whitney *U* and Fisher’s exact test were used for statistical comparisons of continuous and categorical variables respectively.

In stark contrast, seroconversion measured using the same anti‐S antibody assay >14 days after a single vaccination dose in patients with chronic myeloid blood cancers was only 58%, significantly lower than in HCWs of similar age [224/232 (97%) HCW vs 34/59 (58%) patients; *P* < 0·0001; Fig. 1A]. The median anti‐S antibody titre was also significantly lower [630 (IQR 284–1328) vs 75 (IQR 19–328); *P* < 0·0001; Fig 1B].

When split into disease subgroups, seroconversion was highest in patients with CML (75%; Fig. 1C), and observed in 5/6 (83%) of CML patients receiving imatinib (Table  I). There was no difference in seroconversion according to which vaccine was received [22/37 (59%) and 12/22 (55%); *P* = 0·79] for AZD1222 and BNT162b2 respectively (Fig. 1D). While robust interrogation of responses according to treatment regimens was limited by the cohort size, highest response rates (88%, or 7/8 patients) were observed in MPN patients receiving pegylated interferon, but none of the four patients receiving ruxolitinib (MF *n* = 1; ET *n* = 1; PV *n* = 2) and only 36% receiving hydroxycarbamide (4/11 patients; PV *n* = 5; ET *n* = 6) had positive anti‐S serology following the first dose of COVID‐19 vaccination (Table  I). Only 65% (11/17) of patients not receiving any cytoreductive therapy or TKI seroconverted.

There was no difference in the median time from vaccine to anti‐S testing in the patients with positive anti‐S antibodies compared to those without [positive anti‐S: median 34·5 days (IQR 28–56); negative anti‐S median 32 days (IQR 23·5–62·5)], suggesting that the lower rate of seroconversion in the patients with myeloid neoplasms compared to the HCWs was not due to a decline in antibody levels over time.

Tetanus and pneumococcal serology were available for 27 patients and demonstrated immunity in all but one patient. In addition to a complete blood count differential, detailed lymphocyte subset analysis by flow cytometry was performed in 39/59 patients, of whom 15% (6/39) were lymphopenic (lymphocyte count <1·0 × 10^9^/l). Among the lymphopenic patients, 4/6 had a reduction in both absolute CD19 and CD3 cell counts (Table SI). No association was found between absolute lymphocyte count and anti‐S seroconversion [positive anti‐S: median lymphocyte count 1·74 × 10^9^/l (IQR 1·46–2.2); negative anti‐S median lymphocyte count 1·5 × 10^9^/l (IQR 1·1–1.8)].

There is a clear need to protect patients with haematological disorders from COVID‐19 infection and its complications, and to design effective vaccination programmes. This analysis demonstrates that seroconversion rates in patients with chronic myeloid neoplasms are higher than those reported in more aggressive blood neoplasms or those on intensive treatments,^4^ or studies in patients with B‐cell neoplasms.^6^, ^7^ We observed here reasonably high seroconversion rates following a single vaccine dose in patients with CML and in MPN patients receiving interferon, but humoral responses in certain MPN and MDS patients, especially those in patients receiving ruxolitinib and hydroxycarbamide, were found to be substantially impaired as compared to responses in healthy adults of a similar age group. T‐cell‐mediated immunity was not assessed, which may have slightly underestimated the functional response rates to vaccination. Nonetheless, these findings clearly demonstrate an impairment of humoral response. The mechanism for this is not yet known, but is suggestive of both disease‐ and treatment‐mediated immune dysfunction. Although our data show almost ubiquitous presence of anti‐tetanus and pneumococcus antibodies, previous studies in patients with MPNs have also suggested reduced B‐ and T‐cell‐mediated immune responses following vaccination against influenza A strains.^8^, ^9^

The poor seroconversion rates in a group of patients with chronic blood cancers, including those who are not on cytoreductive treatments, those who are in complete haematological remission or major molecular response, suggest a clear need for detailed study and careful interrogation of COVID‐19 vaccination regimens in potentially vulnerable patient groups.^10^, ^11^ Further study with longer‐term follow‐up is required to determine responses to booster vaccine doses, and whether the observation of reduced seroconversion following first vaccine dose will be associated with higher rates of COVID‐19 infection. While it is expected that a higher proportion of patients will respond following booster vaccine doses, the suboptimal responses observed here to the first vaccine dose highlight an unexpected and potentially important immunocompromise in this patient group, which will be informative for planning our ongoing response to this evolving pandemic.

## Funding information

OC is supported by an MRC CARP award. BP is supported by a Cancer Research UK Advanced Clinician Scientist Fellowship and a BRC Senior Fellowship. NS is supported by a CRUK DPhil studentship. LM is supported by a research grant from the Emerson Collective. OC and BP have received ongoing mentorship from the ASH EHA Translational Research Training in Hematology programme. This work was supported by funding from the National Institute of Health Research (NIHR) Oxford Biomedical Research Centre (BRC).

## Author contributions

OC and BP designed the study, collected and interpreted data and wrote the manuscript. HB, GM, NS, KC, CA, MS, CSK and LM collected samples and data, DE, SL and KJ collected data on the HCW and contributed to overall data interpretation, AS, AJM and AP recruited patients and contributed to data interpretation and study design.

## Conflicts of interest

None.

## References

[1] Lee LYW, Cazier J‐B, Angelis V, Arnold R, Bisht V, Campton NA, et al. COVID‐19 mortality in patients with cancer on chemotherapy or other anticancer treatments: a prospective cohort study. Lancet. 2020;395(10241):1919–26. 32473682 10.1016/S0140-6736(20)31173-9 PMC7255715

[2] Williamson EJ, Walker AJ, Bhaskaran K, Bacon S, Bates C, Morton CE, et al. Factors associated with COVID‐19‐related death using OpenSAFELY. Nature. 2020;584(7821):430–6. 32640463 10.1038/s41586-020-2521-4 PMC7611074

[3] Salisbury RA, Curto‐Garcia N, O’Sullivan J, Chen F, Polzella P, Godfrey AL, et al. Results of a national UK physician reported survey of COVID‐19 infection in patients with a myeloproliferative neoplasm. Leukemia. 2021;1–7. 10.1038/s41375-021-01143-2. 33580204 PMC7880652

[4] Monin‐Aldama L, Laing AG, Muñoz‐Ruiz M, McKenzie DR, del Molino del Barrio I , Alaguthurai T, et al. Interim results of the safety and immune‐efficacy of 1 versus 2 doses of COVID‐19 vaccine BNT162b2 for cancer patients in the context of the UK vaccine priority guidelines. medRxiv. 2021:2021.03.17.21253131. 10.1101/2021.03.17.21253131

[5] Eyre DW, Lumley SF, Wei J, Cox S, James T, Justice A, et al. Quantitative SARS‐CoV‐2 anti‐spike responses to Pfizer‐BioNTech and Oxford‐AstraZeneca vaccines by previous infection status. medRxiv. 2021:2021.03.21.21254061. 10.1016/j.cmi.2021.05.041 PMC8180449 34111577

[6] Herishanu Y, Avivi I, Aharon A, Shefer G, Levi S, Bronstein Y, et al. Efficacy of the BNT162b2 mRNA COVID‐19 vaccine in patients with chronic lymphocytic leukemia. Blood. 2021. 10.1182/blood.2021011568 PMC8061088 33861303

[7] Bird S, Panopoulou A, Shea RL, Tsui M, Saso R, Sud A, et al. Response to first vaccination against SARS‐CoV‐2 in patients with multiple myeloma. Lancet Haematol. 2021;8(6):e389–92. 33887255 10.1016/S2352-3026(21)00110-1 PMC8055205

[8] Alimam S, Ann Timms J, Harrison CN, Dillon R, Mare T, DeLavallade H, et al. Altered immune response to the annual influenza A vaccine in patients with myeloproliferative neoplasms. Br J Haematol. 2021;193(1):150–4. 33159465 10.1111/bjh.17096

[9] de Lavallade H , Garland P, Sekine T, Hoschler K, Marin D, Stringaris K, et al. Repeated vaccination is required to optimize seroprotection against H1N1 in the immunocompromised host. Haematologica. 2011;96(2):307–14. 20971824 10.3324/haematol.2010.032664 PMC3031700

[10] Vijenthira A, Gong IY, Fox TA, Booth S, Cook G, Fattizzo B, et al. Outcomes of patients with hematologic malignancies and COVID‐19: a systematic review and meta‐analysis of 3377 patients. Blood. 2020;136(25):2881–92. 33113551 10.1182/blood.2020008824 PMC7746126

[11] Wang Q, Berger NA, Xu R. When hematologic malignancies meet COVID‐19 in the United States: infections, death and disparities. Blood Rev. 2021;47:100775. 33187811 10.1016/j.blre.2020.100775 PMC7833659

