# Supplementary material for: Impaired antibody response to COVID‐19 vaccination in patients with chronic myeloid neoplasms
Source: Br J Haematol. 2021 Jun 24;194(6):1010–5. doi: 10.1111/bjh.17644 (PMC8444839; doi:10.1111/bjh.17644)
Supplement: Supplementary file 1 [file BJH-194-1010-s001.docx]

Suppl. Table 1: Extended haematological characteristics of patient cohort with lymphocyte subsets.
